# Conflict management and communications skills in the ICU: a qualitative study on forming junior critical care nurses through drama pedagogy

**DOI:** 10.1186/s12912-026-04487-1

**Published:** 2026-03-02

**Authors:** Sepideh Olausson, Susanna H. Arveklev, Frances Lin, Wendy Chaboyer, Mona Ringdal

**Affiliations:** 1 Institute of Health Care Sciences, Sahlgrenska Academy, University of Gothenburg, Gothenburg, Sweden; 2 Region Västra Götaland, Sahlgrenska University Hospital, Department of Anaesthesiology and Intensive Care, Gothenburg, Sweden; 3 Department of Health Sciences, University West, Trollhättan, Sweden; 4 Caring Futures Institute, College of Nursing and Health Sciences, Flinders University, Adelaide, Australia; 5 Griffith University, Gold Coast Campus, Gold Coast, QLD 4222 Australia

**Keywords:** Critical care, Drama, Education, Focus groups, Learning method, Non-technical skills, Communication, Teamwork

## Abstract

**Background:**

Working as a nurse in intensive care units (ICUs) necessitates the possession of technical and non-technical skills in providing high-quality care to critically ill patients. However, current critical care curricula often do not offer training in such skills. To address this gap, drama workshops were developed in this study to improve non-technical skills in post-graduate critical care nursing education.

**Aim:**

We aimed to understand critical care nursing students’ perceptions of the drama programme, which focused on communication, teamwork and conflict management in the ICU.

**Methods:**

Focus groups and one individual interview with 19 participants recruited from a post-graduate (or master’s) critical care programme were conducted. Thematic qualitative analysis was used to analyse the data.

**Findings:**

Three themes were identified: Personal and professional development, Emotional engagement and psychological safety in learning, Struggling to maintain the professional identity and role performance. The findings suggest an increased awareness of teamwork and individual responsibilities. Participants described an increased self-awareness, particularly regarding body language and emotional responses during interpersonal conflicts. They valued the opportunity for reflection and peer discussion, which deepened their understanding of team dynamics. However, performance anxiety, especially when portraying unprofessional roles, and the lack of an authentic environment were identified as barriers to engagement.

**Conclusion:**

The findings suggest that drama-based pedagogy can serve as an effective medium for facilitating reflective engagement and the development of non-technical professional competencies, particularly in conflict management. These findings point to the value of embodied, experiential approaches in higher education curricula aimed at fostering deeper and affective learning processes and a complement to simulation-based teaching.

**Implications for clinical practice:**

Teamwork in the ICU can benefit from drama-based learning programmes as it increases awareness regarding the significance of collaboration within the healthcare team. Moreover, it can help develop nurses’ non-technical skills, which are essential in caring for critically ill patients and initiating effective interprofessional interactions.

**Clinical trial number:**

N/A.

## Introduction

Intensive care units (ICUs) provide essential life support for critically ill patients. This requires nurses to develop strong clinical and professional competence through their education [1]. ‘Clinical competence’ refers to the ability to identify and respond to abnormal conditions in a patient’s health and ensure appropriate care [2]. In ICU achieving clinical competence entails meeting three key requirements: compliance with clinical regulations, adherence to nursing ethics principles and implementation of effective nursing interventions [1]. Providing safe and effective ICU care requires, therefore, both technical skills (e.g., airway suctioning) and non-technical skills, such as teamwork, communication, critical thinking, and managing stressful situations. These skills are essential to professional competence and patient safety, particularly for making informed decisions about patient care [3, 4]. However, when they are underdeveloped, communication breakdowns and interpersonal conflict can arise in ICU teams. A multi-centre survey study with 5,268 ICU clinicians from 323 ICUs in 24 countries showed that 70% of ICU staff perceived conflicts, most commonly between nurses and physicians, followed by intra-nursing and staff–relative conflicts [5]. Strong communication and conflict management skills are essential not only in maintaining teamwork with fellow staff but also for engaging with patients and their families. Although these topics are often addressed theoretically in curricula, there is limited emphasis on experiential learning or skills-based training, particularly at the post-graduate level for critical care nurses (CCNs). Yet current critical care education often lacks practical training in this area [6]. To address this gap, we developed and evaluated an intervention to improve nursing students’ non-technical skills using drama workshops. The programme focuses on communication and teamwork including conflict management and can be used in post-graduate specialised CCN education.

Nursing education, including post-graduate studies and specialisation pathways, varies across countries as they implement distinct nursing regulations and educational frameworks. This contributes to variations in the scope of practice, education and training of nurses. Many European countries offer post-graduate education for nurses to become certified CCNs. However, having a critical care diploma or certificate is not always required for working in ICUs [7].

Simulation-based learning is a well-established pedagogical approach in postgraduate intensive care nursing education and is commonly used to support the development of clinical competence and non-technical skills through scenario-based training and structured reflection [8]. However, simulation is typically organised around predefined clinical scenarios, time pressure and performance goals, which may limit opportunities for sustained exploration of interpersonal dynamics and conflict situations. Drama pedagogy is a complementary approach, providing a less performance-driven learning environment focused on communication, teamwork and conflict management. Although drama-based learning remains uncommon in intensive care nursing education, research in nursing education shows that using drama in education is an effective pedagogical strategy, supporting the development of ethical, relational, and communication skills among students [9]. By engaging in drama activities, nursing students interact with peers, adopt different roles, and explore diverse perspectives, which can enhance self-awareness and empathy [10]. Drama also offers a dynamic way to develop conflict management skills which is essential for ICU settings. A critical review found that drama-based learning improved students’ interpersonal skills, understanding of patient experiences, professional identity, communication, self-reflection, and critical thinking [11]. However, participation can be hindered by students feeling discomfort or unfamiliarity with drama methods, which may limit their engagement and the overall effectiveness of the learning experience [10, 12]. While most existing studies have focused on drama as a teaching method in undergraduate nursing programmes, few have examined the use of drama in post-graduate education, particularly in critical care settings. Therefore, this qualitative study aimed to explore CCN students’ perceptions of a drama programme focusing on communication, teamwork, and conflict management in the ICU.

## Methods

We employed a qualitative descriptive research design [13, 14] using focus groups and interviews to explore students’ perceptions of a drama programme. Our ontological orientation lies within the constructivism, acknowledging existence of multiple realities that are socially constructed [15]. Epistemologically, constructivism holds that knowledge is produced through interpretive processes embedded in social, cultural, and historical contexts, where meanings are co-constructed by the subjects/individuals rather than objectively discovered [15].

Focus groups involve gathering data through group discussions centred around pre-determined topics using a structured or a semi-structured design [16, 17]. Their primary objective is to elucidate individual and collective perspectives of the phenomenon under study [16, 17]. However, only one student participated in one of the data collection sessions, resulting in an individual interview. We followed the consolidated criteria for reporting qualitative research (*COREQ*) guidelines in reporting the study [18].

### Drama workshops

The drama workshops were part of a Master’s programme in Critical Care, designed to help students develop non-technical skills in conflict management, teamwork, and communication within the ICU setting. The programme was developed in collaboration with the critical care education team and drama pedagogues. During the workshops, the drama pedagogues facilitated the drama exercises, while the lecturers led the reflective discussions. The learning objectives addressed in the drama workshops included (1) Critically reflecting on personal and professional interactions with patients, relatives, and members of the healthcare team. (2) Analysing and reflecting on nurses’ ethical, moral, and legal responsibilities, as well as their professional accountability within healthcare settings.

Two workshops were delivered and led by a drama pedagogue and a senior lecturer in critical care. Each drama workshop had three phases: (1) Framing and warm-up; (2) Drama exercise; (performing the scenarios); (3) Reflection and discussion. The first workshop was held during the first three weeks of the post-graduate CCN programme. It provided an orientation in the drama technique with pre-prepared ICU conflict cases featuring miscommunication and conflicts with patients, relatives and healthcare personnel in the ICU. It lasted approximately three hours. The second workshop was conducted after students had completed the 8-week clinical training in the ICU, approximately halfway through the programme. One 8-hour day was used to follow up on miscommunication and conflict management in the ICU using drama pedagogy. The students were asked to discuss a case about miscommunication or conflicts they witnessed during their clinical training, and then a drama workshop was performed based on the cases they observed.

### Setting and sample

The study employed a purposeful sampling strategy [19]. We invited students from a 40-week CCN programme, full-time Master’s in Nursing Science programme offered at Gothenburg University in Sweden to participate in the study. All the enrolled students had participated in the drama workshop as a mandatory part of their education. The inclusion criterion was being enrolled in the course, so all students (*n* = 60) were invited to participate. The participation in the study was voluntarily and, a total of 19 CCRN students accepted the invitation to participate in this study.

### Data collection

Data were collected through six focus group interviews (3–5 students per group) and one individual interview with CCRN students after the final workshop. The participants were provided with oral and written study information, and their voluntary participation was stressed. Focus groups were conducted by [SO] and [SHA], who were not involved in the drama workshops. The interview guide was developed based on the literature, with input from critical care and drama experts. The focus groups held in the university’s conference room were scheduled during lunch breaks when the students had campus-based activities. The participants were offered lunch and beverages. The focus groups started with a short presentation of the study’s main aim. Thereafter, the participants were invited to introduce themselves and cite their previous experiences as nurses and whether they had already experienced drama workshops. The interview guide consisted of the following questions:

- Can you tell us about any drama workshop experiences during your studies?
- How was your experience playing various parts in the drama workshops?
- What have you learned about conflict management, teamwork and communication?
- Which part of the workshop was valuable for your learning and understanding of the topics discussed?
- Is there anything we can do to improve the workshop?

Prompt questions (e.g. ‘Can you please tell us more?’ ‘Can you please give us an example?’ and ‘Do you all agree on this or is there anyone who thinks differently?’) were raised as needed. The interview guide did not evolve during the study. The focus groups lasted between 60 and 90 min, while the individual interview lasted 60 min. The interviews were transcribed verbatim. The researchers conducting the focus groups did not participate in the drama workshops.

### Data analysis

Data were analysed through an inductive content analysis described by Elo and Kyngäs [20]. The analytical process is characterised by a dialectical pattern that moves from the whole to the parts, generating a ‘new whole’ in the process. The analysis was conducted in three phases: preparatory, coding and reporting. First, the data were collected, transcribed and analysed in Swedish. The Swedish members of the research group started the preparatory phase by reading each interview text carefully to gain an understanding and sense of the whole text. We also identified meaning units consisting of text segments corresponding to the aim of the study and the subject matter. These units were then subjected to open coding (i.e., systematic grouping of the codes by the contents and labelling them) done by the first author and confirmed by SHA and MR. Thereafter, we critically examined the codes by posing questions to the text, such as ‘What does this mean?’ and ‘How can I be sure of that?’ Our intention was to identify the related meanings that corresponded to the aim of the study. The meanings were compared against each other and against the interviews, generating overall themes as conceptualised by the reporting phase Elo and Kyngäs [20]. The non-Swedish-speaking researchers participated by re-examining the emerging findings once they were translated into English, helping refine the analysis.

### Rigour

To ensure credibility and establish trustworthiness, the authors used several strategies: we called our pre-understanding into question, used an audit trail and discussed our findings with peers at a scientific seminar. The findings were carefully reviewed by all the authors, and amendments were made prior to finalisation. Moreover, the authors exercised reflexivity by using a critical approach during the whole process of the research to establish trustworthiness. Authors, WCH, FL, MR and SO are all critical care nurses and researchers in the context of intensive care as well as experts in qualitative methodologies. Author SHA holds a PhD in drama pedagogy and experienced in qualitative and pedagogical research.

### Ethical considerations

Oral and written information about the study was provided, in which students were informed that they could withdraw at any time and that any decision not to participate would have no consequences. Written consent to participate in the study was obtained from all the participants before collecting data. The researcher who conducted the interviews (SO and SHA) did not participate in the workshops. The project was approved by the Swedish Regional Board of Ethics in Gothenburg (D no: 371 − 16).

### Findings

A total of 19 post-graduate critical care nursing students participated in the study through focus group interviews, and one individual interview, including 18 female students and one male student. Participants’ previous working experience as registered nurses ranged from 2 to 10 years, and seven participants reported prior experience of drama pedagogy. The analysis resulted in three overarching themes, each comprising two sub-themes, reflecting students’ experiences of participating in drama-based workshops focused on communication, teamwork, and conflict management in the ICU context.

### Personal and professional development

#### Gaining new understandings through reflection

A central theme that emerged was participants’ personal and professional development through reflection and elevated self-awareness. Participants described a growing recognition of the critical role of teamwork and individual responsibility in managing conflicts and navigating complex clinical situations. *“For me*,* personally*,* I don’t think that it was the drama itself*,* but it is…when we analysed what we saw” (FG3)*.

They reflected on the significance of body language in communication with patients, families, and teammates, noting that non-verbal cues often shaped the emotional tone and effectiveness of their interactions. Reflection during and after the workshops led to fruitful insights into different interpretations and analyses of the conflicts.

*Hm you know*,* body language. There are other ways to demonstrate it as well. We could lean forward or lean back … or look in a different direction. It seems that these actions contribute more to the learning process than just relying on understanding body language (FG6)*

#### The power of reflection and self-awareness

Several participants described how this reflective process helped them uncover unconscious assumptions and biases that may influence their reactions in delicate situations. By reflecting on their own views alongside those of others, participants came to better understand the nuanced and often complex nature of the situations they encountered. For example, some participants realized that what initially appeared to be a straightforward disagreement was often rooted in miscommunication or differing expectations.

*I think you really learn something from that…getting to feel for yourself just how difficult it is to talk to someone who isn’t interested. It’s not something you’d ever want to put someone else through. (FG4)*

Participants’ previous nursing experience appeared to shape how they engaged with the drama pedagogy. Those with more clinical exposure sometimes relied on habitual responses, which could both enrich their insights and limit their willingness to embrace unfamiliar perspectives.

These responses could create a challenge, as participants found it difficult to identify and critically reflect on their own assumptions, sometimes mistaking them for personal faults or limitations.

*It feels like this should already be somewhat a common knowledge; that one should be mindful of not appearing closed off and maintaining eye contact. It’s like if someone hasn’t realised this in their profession as a nurse*,* they might have missed an important aspect of their work. (FG1)*

While the participants did not gain explicit or step-by-step conflict management strategies, they described developing greater self-awareness and increased mindfulness in their future approaches to conflicts.

*No*,* I wouldn’t say I’ve seen it as a specific learning tool*,* but certainly as a form of reflection and I think that kind of reflection is very valuable* (FG 2)

### Emotional engagement and psychological safety in learning

#### A creative learning environment

Participants shared descriptions of their experiences of the workshop environment itself. Many expressed that they found the sessions enjoyable, fun, and emotionally engaging. They embraced the format, expressed enthusiasm, and actively participated. The workshops were found facilitating a playful transition between seriousness and humour while addressing the dilemmas presented in the different scenarios.

*You dare to be yourself at least*,* I mean. You’re allowed to laugh at the same time as things are serious; even though the situations and topics we touch on are serious*,* you still have to be allowed to laugh. That’s why I think these kinds of drama workshops can be good…. there’s a very short distance between laughter and tears. They’re very different emotions*,* shifting quickly from one to the other (GF 1)*

This provided a psychologically safe space where participants could experiment with different responses to conflicts without fear of judgment. The active involvement of the entire group enhanced the exploration of each scenario, fostering a sense of collective learning and mutual support.

#### A catalyst for learning

Participants also reflected on the structure of the workshops, particularly the time allocated for reflections and discussions following each session. These were perceived as more meaningful than the acting itself. Engaging in the drama activities served as a catalyst for these reflections, wherein conflict situations and teamwork became embodied and could be experienced from new perspectives. However, while these reflections were valued, participants emphasized that they did not equip them with concrete skill acquisition.

*Well*,* that’s actually what I find a bit difficult to put into words right now*,* but if I say that learning*,* for me*,* is more (pause*,* breathe)*,* perhaps there is a guiding aspect to it. However*,* this drama pedagogy or workshop didn’t direct us. It didn’t tell us what was right. It didn’t explain or show the way*,* not at any point*,* not even in the end*,* and for me*,* that’s probably part of learning something. It somehow requires a guide*,* whether it’s a teacher*,* a mentor or a supervisor. Someone who says*,* ‘Here are the rules*,* and they apply’. It includes someone guiding me to some extent. I think that’s possibly why I haven’t thought of it as learning*,* but more as reflection*,* as I mentioned earlier. Nevertheless*,* it’s still valuable*,* of course*,* to reflect. (IP1)*

The discussions helped clarify complex situations and reveal behavioural patterns, but participants felt that the brief exposure (only two workshops) was insufficient for meaningful transformation.

### Struggling to maintain the professional identity and role performance

#### Challenges to engaging with drama workshops - Internal barriers

While acknowledging the advantages of engaging in drama and recognising its potential to provide tools for reflection and conflict management, the students described several internal barriers that affected their engagement. Central among these was a sense of pressure to perform and act, emphasising the need to adopt a role and be someone other than themselves. This pressure can be seen as an internal tension which was further amplified by participants’ perception that the conflict situations were exaggerated, despite being rooted in cases derived from authentic clinical cases (first workshop) and their own observations (second workshop).

*I think discussions would be much better*,* and you can see and reflect oh…it is this one…[I] can approach this problem. Drama requires something of me that I can’t give. (FG5)*

The participants stressed that joining a drama workshop without prior acquaintances with fellow students triggered insecurity and vulnerability. This was evident during the first workshop session, when the group members were still unfamiliar with one another. As a result, their attention was internally divided between the workshop itself and their concerns about being observed, judged, and not meeting perceived performance expectations. This created challenges in terms of openly sharing reflections and maintaining their focus on the workshop’s content.

*You know*,* this is really a personal thing to stand there and play the scenario… I really don’t think people should be forced. It doesn’t fit everybody…my personality. I thoroughly believe it should be voluntary… (FG6)*

What appeared to be specifically challenging for them was the fact that they sometimes had to play the role of ‘a bad nurse’. They expressed discomfort in acting out inappropriate behaviour, which conflicted with their desired professional identity. They felt the need to justify their actions during role play, indicating a struggle to protect their professional self-image, which did not align with how they wished to be perceived in their profession. This meant the un-dignification of their professional self-esteem and, therefore, a shifting focus from learning and reflecting to *performing* why the essence of drama education was lost.

*If I’m playing an arrogant person*,* I have to do it with my whole body language; otherwise it shows that I’m not really in the role. So yes*,* you do look at the other people as well*,* I definitely think so // I find it uncomfortable to have to look angry or distressed…. I don’t enjoy it. I’m not like that; I don’t want to look angry or be rude. What happens to you*,* sort of*,* or what is it that’s happening? There are so many other ways to deal with things. I don’t want to look angry or distressed; I feel like it works itself out anyway in the situation I’m supposed to portray. It’s about the work—it has nothing to do with me as a person or who I am. (FG 2)*

#### Challenges in creating an authentic environment - External barriers

One recurring theme that emerged from the data was the participants’ desire for an authentic environment, specifically an ICU patient room, highlighting external and contextual barriers related to the workshop design and setting. *”Here we are*,* four walls and a circle of chairs…this is not a natural environment for us*,* we don’t work here*,* and this is not a place where we nurse patients”. (FG2)*

There were frequent discussions about feeling uncomfortable when conducting drama workshops in a classroom setting, which was perceived as lacking resemblance to and the situational safety of an actual ICU environment. This discrepancy was identified as an obstacle to fully understanding the purpose of the workshops. The students mentioned the simulation centre as a more realistic environment with which they were familiar and a place they associated with a sense of clarity regarding certain roles, tasks, learning objectives and expectations. They compared the drama workshops to other learning activities, for example simulation, in the critical care programme, expressing that the workshops felt too abstract and that this required a greater need for theoretical understanding.

*I think it also needs to be seen in the context of the rest of the training*,* which often deals with really acute and traumatic or even sudden death. Then*,* shifting into a drama exercise that’s very lighthearted can feel a bit pointless like it lacks depth or meaning. (FG3)*

Furthermore, some of the participants suggested that incorporating professional actors/actresses would be more beneficial, particularly if they portrayed patients or family members, while the students took on the role of nurses during the drama sessions. These modifications were believed to enhance the authenticity of the workshops, reduce ambiguity in role expectations, and thereby creating a deeper sense of reality.

## Discussion

In this study, we sought to explore post-graduate CCN students’ experiences of a drama programme focused on communication, teamwork and conflict management in the ICU. Our findings revealed that drama workshops can have a positive impact on both professional and personal growth, facilitating students’ skills in conflict management, teamwork and communication. However, they also presented challenges as students felt pressured to focus more on acting and performing rather than being present in the process of drama and reflection. Furthermore, the environment in which the workshops took place, a traditional classroom, posed a significant challenge in terms of creating a sense of safety and authenticity. This prevented participants from fully engaging and connecting with the content of the workshops.

To the best of our knowledge, this is the first study examining post-graduate CCN students’ experiences of a drama programme in the context of critical care education, as well as its contribution to the development of nurses’ non-technical skills. Such skills consist of social (teamwork, leadership and communication), cognitive (situational awareness, decision-making, cognitive readiness and task management) and personal management (stress and fatigue management) skills necessary for safe and effective performance [21].

One aspect in which we believe the drama workshops made a significant contribution is conflict management. CCN students gained new insights into their responsibilities as team players and discovered new ways to approach conflicts and their possible interpretations. This finding is consistent with those of previous studies [9, 22]. Arveklev et al., [9] demonstrated that drama has the potential to enhance non-technical skills for nursing students, particularly communication skills. Similarly, our findings confirm the result of Uzunoz and Demirhan [22] that drama in higher education has the potential to increase critical thinking and students’ openness and ability to initiate divergent thinking.

We also found that drama activities were a powerful tool in enhancing CCN students’ learning through reflection. A concept analysis of reflection in nursing professional development [23] describes the essence of reflection with a four-fold pattern, thereby filling the gap between theory and practice, expanding the role of nursing and enhancing competency, and addressing nurses’ educational and learning needs and responsibilities.

Prior to the drama workshops, the students had participated in several simulation exercises, where reflections were held as debriefing sessions after the simulations. Reflection during debriefing in simulation-based learning is described as essential for consolidating non-technical skills through structured post event reflection, which Schön [24] calls reflection-on-action [25]. However, Schön emphasizes reflection-in-action, adapting in real time under uncertainty; Rolfe [26] further argues that nursing education often overemphasizes retrospective reflection at the expense of in the moment adjustment. Drama, on the other hand, fosters this by offering the opportunity to reflect both in- and on-action [12]. In this study, this became apparent when students described how they were forced to reflect about their own reactions, in-action, while acting in role during the dramatized situations. For example, some took on challenging roles that felt far from their natural responses, prompting them to confront emotional and behavioural contrasts. By stepping into these roles, observing their peers, and participating in both self-reflection and group discussions, students engaged in a learning process that aligns with Dewey’s “learning by doing” [27] and Kolb’s experiential learning cycle, which integrates concrete experience, reflective observation, abstract conceptualization, and active experimentation [28].

Moreover, from a philosophical perspective, reflection is linked to the two overarching themes of ‘knowing’ and ‘not knowing’ [29]. In other words, reflection involves learning from one’s experiences based on a process of internalising and processing emotions and reactions, which leads not only to formulating other strategies for the future but also to being aware of one’s lack of knowledge, i.e. being alert and curious in situations [23, 29]. This indicates a transformation of one’s perspective with a critical attitude and openness. When students acquire these qualities/skills, they are better equipped to deliver high-quality care for critically ill patients and their families.

Our findings showed that CCN students experienced some pressure and discomfort in meeting performance expectations, particularly when playing the role of a bad or unprofessional nurse. Speculating whether these feelings stemmed from shyness or were perceived as conflicting with their professional identity remains challenging. However, even individuals who are generally shy can benefit from engaging in drama because assuming a new role and envisioning themselves as someone else can facilitate personal growth [30]. Nevertheless, the resistance felt by the CCN students in our study was more likely associated with their perceptions of the professional image expected of a competent nurse. This is supported by statements from several participants who expressed a preference for being ‘themselves as a nurse’ in the scenarios. Behaving unprofessionally and displaying inappropriate conduct are widely regarded as unacceptable by most nurses [31]. Thus, it is crucial for CCN students to acknowledge this fact, as such behaviour can have a harmful impact on staff, patients and the entire organisation [31].

Furthermore, we observed that the CCN students were more used to participating in simulation workshops that emphasised clinical skills training. They lamented a lack of an ‘authentic environment’ during the drama workshop and did not feel entirely safe. In simulation workshops, the creation of an authentic environment is highly significant. While drama has numerous benefits for nursing education in non-technical skills, simulation appears to be the optimal approach for teaching technical skills, such as vital signs assessment [11]. Based on our findings, it is suggested that incorporating authentic environments into drama workshops for students could be advantageous.

Finally, our findings both confirm and contradict a previous study on learning through drama in a specialist post-graduate nursing programme in paediatric care by [12]. Their findings indicated two different perspectives among students: some preferred active participation in the drama workshops, while others preferred a more passive role by observing their peers in the scenarios. Furthermore, while the students in the study of Arveklev et al., [12] expressed that drama facilitated their learning and preparedness for their professional role, our study found conflicting views. In particular, while some CCN students in our study preferred passive rather than active participation, they still acknowledged the valuable knowledge gained from the drama workshop.

### Strengths and limitations

This study has several strengths. First, the focus groups were facilitated by two researchers who were independent of those responsible for conducting the drama workshops. We believe this strategy minimized the risk of perceived pressure on students, thereby enabling them to feel comfortable and share their experiences freely. At the same time, we acknowledge that social, relational, or contextual biases, reflecting inherent power dynamics, may still have influenced participants in ways that cannot be fully controlled.

Additionally, the students displayed a genuine interest in participating in the interviews, and their responses exhibited diverse perspectives. The decision to use focus groups not only deepened our understanding of the value of incorporating drama in critical care education but also emphasised the importance of tailoring a programme suited to the critical care curriculum, ensuring its meaningfulness for the CCN students.

One limitation of focus groups is the potential risk of groupthink, which cannot be entirely ruled out in this study. The participants may have been influenced by the opinions of others, potentially hindering the full disclosure of individual perspectives. Although an individual interview was conducted as part of this study, it was not initially planned. We decided to include the one individual interview along with focus groups as the interview provided rich and detailed information on drama pedagogy and was believed to enrich our data set. This individual data was analysed together with the focus groups and treated in the same way.

Moreover, despite utilising an interview guide, there was the possibility of unintentionally influencing the discussion [16, 17]. Additionally, we see a risk that students who chose to participate in the study may have been more open to reflective or creative pedagogical approaches. This may have influenced the overall positive tone of the findings and introduced selection bias. Moreover, the transferability of the finding may be limited to the post graduate programme in Critical Care and to Swedish or Scandinavian educational context and not directly adaptable to other countries. Nevertheless, we gained valuable insights into the CCN students’ experiences of participating in drama workshops and their learning processes and this may be an inspiration to develop teaching strategies addressing non-technical skills.

## Conclusions

We found that the drama method was beneficial in engaging students in a fun and creative way in their learning. A focus on reflections during the drama workshops enabled a deeper understanding of how to resolve conflicts, leading to increased clarity and the emergence of valuable insights that facilitated transformative learning experiences. Based on our findings, when using the drama method in education, a clear alignment between the drama programme and the desired learning outcomes must be established. In addition, future research should consider to (1) increase number of workshops throughout the CCNs’ education to consolidate their learning and improve effectiveness; (2) conduct the workshop in a simulated environment instead of the classroom we used; and (3) timing of the drama method later stage of the students study when they feel safer to share their thoughts and feelings in a group. Overall, drama workshops have shown the potential in our study to enhance the development of non-technical professional competences among CCN students, particularly in areas such as conflict resolution training.

## Data Availability

De-identified excerpts from the focus group transcripts are available from the corresponding author upon reasonable request. Full transcripts are not publicly available due to confidentiality agreements with participants.

## Declarations

### Author note

This study is part of a larger collaborative international programme (Erasmus Plus) between University of Gothenburg, Sweden and Griffith University, Australia, entitled “Drama in Intensive and Acute Care, DIAC”.
